# Challenges and a call to action for protecting European red wood ants

**DOI:** 10.1111/cobi.13959

**Published:** 2022-08-05

**Authors:** Paride Balzani, Wouter Dekoninck, Heike Feldhaar, Anne Freitag, Filippo Frizzi, Jan Frouz, Alberto Masoni, Elva Robinson, Jouni Sorvari, Giacomo Santini

**Affiliations:** ^1^ Department of Biology University of Florence Sesto Fiorentino Italy; ^2^ Faculty of Fisheries and Protection of Waters, South Bohemian Research Center of Aquaculture and Biodiversity of Hydrocenoses University of South Bohemia in České Budějovice Vodňany Czech Republic; ^3^ Royal Belgian Institute of Natural Sciences Brussels Belgium; ^4^ Department of Animal Ecology I, Bayreuth Center of Ecology and Environmental Research (BayCEER) University of Bayreuth Bayreuth Germany; ^5^ Cantonal Museum of Zoology Lausanne Switzerland; ^6^ Institute for Environmental Studies, Faculty of Science Charles University Prague 2 Czech Republic; ^7^ Department of Biology University of York York UK; ^8^ Department of Biology University of Turku Turku Finland; ^9^ Natural Resources Institute Finland (Luke) Helsinki Finland

**Keywords:** conservation target, forest diversity, *Formica rufa* group, insects, legislation, red wood ants, diversidad forestal, grupo *Formia rufa*, hormiga roja de la madera, insectos, legislación, objetivo de conservación, 昆虫, 红褐山蚁, 红褐山蚁 (*Formica rufa*) 群, 森林多样性, 保护目标, 立法

## Abstract

Red wood ants (RWAs) are a group of keystone species widespread in temperate and boreal forests of the Northern Hemisphere. Despite this, there is increasing evidence of local declines and extinctions. We reviewed the current protection status of RWAs throughout Europe and their International Union for the Conservation of Nature (IUCN) threat classification. Only some RWA species have been assessed at a global scale, and not all national red lists of the countries where RWAs are present include these species. Different assessment criteria, inventory approaches, and risk categories are used in different countries, and data deficiency is frequent. Legislative protection is even more complex, with some countries protecting RWAs implicitly together with the wildlife fauna and others explicitly protecting the whole group or particular species. This complexity often occurs within countries, for example, in Italy, where, outside of the Alps, only the introduced species are protected, whereas the native species, which are in decline, are not. Therefore, an international, coordinated framework is needed for the protection of RWAs. This first requires that the conservation target should be defined. Due to the similar morphology, complex taxonomy, and frequent hybridization, protecting the entire RWA group seems a more efficient strategy than protecting single species, although with a distinction between autochthonous and introduced species. Second, an update of the current distribution of RWA species is needed throughout Europe. Third, a protection law cannot be effective without the collaboration of forest managers, whose activity influences RWA habitat. Finally, RWA mounds offer a peculiar microhabitat, hosting a multitude of taxa, some of which are obligate myrmecophilous species on the IUCN Red List. Therefore, RWAs’ role as umbrella species could facilitate their protection if they are considered not only as target species but also as providers of species‐rich microhabitats.

## Resumen

Las hormigas rojas de la madera (HRM) conforman un grupo de especies clave con amplia distribución en los bosques templados y boreales del Hemisferio Norte. A pesar de lo anterior, cada vez hay más evidencia de su declinación y extinción local. Revisamos el estado actual de protección de las HRM en toda Europa y su clasificación en la Lista Roja de la Unión Internacional para la Conservación de la Naturaleza (UICN). Sólo se han evaluado algunas especies de HRM a escala mundial y no todas las listas rojas nacionales de los países con presencia de HRM incluyen a estas especies. Los diferentes países usan criterios de evaluación, estrategias de inventario y categorías de riesgo distintos, además de que la información deficiente es habitual. La protección legislativa es todavía más compleja pues algunos países protegen implícitamente a las HRM junto con la fauna silvestre y otros protegen explícitamente a todo el grupo o a una especie particular. Esta complejidad ocurre a menudo en los países (por ejemplo: Italia) en donde, fuera de los Alpes, sólo se protege a las especies introducidas, mientras a las especies nativas, que están declinando, no se les protege. Por lo tanto, se requiere un marco de trabajo internacional y coordinado para proteger a las HRM. Esto necesita primero que se defina el objetivo de conservación. Ya que las HRM tienen similitudes morfológicas, una taxonomía compleja e hibridación frecuente, la protección del grupo completo, con la distinción entre las especies autóctonas y las introducidas, parece ser una estrategia más eficiente que la protección de una sola especie. Segundo, se debe actualizar la distribución actual de las HRM en Europa. Tercero, una ley de protección no puede ser efectiva sin la colaboración de los gestores forestales, cuya actividad influye sobre el hábitat de las HRM Finalmente, los montículos de las HRM ofrecen un microhábitat peculiar pues hospedan a una multitud de taxones, algunos de los cuales son especies mirmecófilas obligadas presentes en la Lista Roja de la UICN. Así, el papel de las HRM como especie paraguas podría facilitar su protección si se les considera no sólo como especies diana sino también como proveedoras de microhábitats con riqueza de especies.

## 【摘要】

红褐山蚁是广泛分布于北半球温带及寒带森林中的一群关键物种。尽管如此, 有越来越多的证据表明红褐山蚁正在发生局部种群下降和灭绝。本研究回顾了整个欧洲的红褐山蚁目前的保护状况及其在世界自然保护联盟(IUCN)的威胁分类。我们发现, 只有部分红褐山蚁物种得到了全球范围内的评估, 而且并非所有存在红褐山蚁分布的国家的红色名录都包括这些物种。不同国家使用不同的评估标准、清查方法和风险类别, 而且经常出现数据缺乏的情况。立法保护则更为复杂, 一些国家的红褐山蚁保护隐含在野生动物群保护之中, 而另一些国家则明确地保护整个群体或特定物种。这种复杂性经常发生在国家内部, 例如在意大利, 在阿尔卑斯山以外的地方, 只有引进种得到保护, 而正在减少的原生种却没有得到保护。因此, 需要一个全球合作框架来保护红褐山蚁。这首先需要确定保护目标。由于形态相似, 分类复杂, 杂交频繁, 保护整个红褐山蚁群似乎是比保护单一物种更有效的策略, 尽管需要区分原生种和引进种。第二, 需要更新红褐山蚁物种在欧洲的分布情况。第三, 保护法发挥作用还需要森林管理者的合作, 因为他们的活动会影响红褐山蚁的栖息地。最后, 红褐山蚁土丘提供了一个特殊的微生境, 容纳了许多类群的物种, 其中一些是《IUCN濒危物种红色名录》中的专性适蚁物种。因此, 如果不仅将红褐山蚁视为目标物种, 同时还将其视为多物种微生境的提供者, 那么红褐山蚁作为伞护种的作用将进一步促进其保护。**【翻译:胡怡思;审校:聂永刚】**

## INTRODUCTION

With at least 13 species described in the Palearctic and up to 19 species reported in North America, red wood ants (RWAs) (i.e., species belonging to the *Formica rufa* group) are ecologically dominant species (Stockan et al., 2016). These ants are considered keystone species in temperate and boreal forests of Eurasia. Due to their large and long‐lasting nests, they affect the functioning of mainly forest ecosystems in many ways and across several trophic levels (e.g., by controlling forest pest species) (Trigos‐Peral et al., 2021). Although RWA species are still abundant in many parts of their distribution range, their conservation raises increasing concerns (Breen, 2014; Cherix et al., 2012; Dekoninck et al., 2010; Mabelis & Korczyńska, 2016). Indeed, there is evidence of local decline or even extirpation. For example, *F. uralensis* was extirpated from Switzerland (Cherix & Maddalena‐Feller, 1986), whereas the scattered relict populations of this species in France, Germany, and Poland are at high risk of extirpation (Stankiewicz et al., 2005; Wegnez & Mourey, 2016). Moreover, local information is scattered and sometimes contradictory. For example, *F. pratensis* is reported as extinct in mainland Britain since at least 1988 (Nicholson, 1997). However, its presumed extinction is frequently erroneously dated to 2005, the year of the last update for this species on the Bees Wasps and Ants Recording Society (BWARS, www.bwars.com), although the page reports, “The last known nest, near Wareham, died out in 1987.”

The main threats to these species have been discussed in detail by Sorvari (2016). However, it is worth stressing that the relative importance of these threats varies considerably in different parts of their Palearctic distribution range. In the southernmost countries, RWAs are restricted to mountain areas, whereas in their northernmost range, they also occur at lower elevations (Stockan et al., 2016); threatening factors may thus differ. Additionally, their problematic taxonomy, with some species identifiable only through molecular analysis (Bernasconi et al., 2010), the presence of cryptic species (Bernasconi et al., 2011; Seifert, 1996, 2021), and widespread hybridization (Beresford et al., 2017; Seifert et al., 2010), makes it difficult to efficiently assess population size and distribution.

Despite their ecological importance and widespread distribution, Hymenoptera, with the exception of wild bees (Drossart & Gérard, 2020; Kleijn et al., 2015), lag behind other insect taxa, like Lepidoptera or Coleoptera, as conservation targets (Leandro et al., 2017). Ants (particularly RWAs) were an early group to be defined as vulnerable and worthy of protection (Wells et al., 1983). Given the importance of RWAs in forest pest management, the European Council recommended as early as 1965 that all the member states adopt legal provisions for protecting these species, highlighting their decline and the need for their conservation (Pavan, 1981). However, more than 50 years later, there is no unique legal framework, and contradictory measures are sometimes taken. The importance of the focus on RWA protection extends beyond the conservation of these species per se. They are important ecosystem engineers and umbrella species (e.g., Balzani et al., 2021a), so their conservation is relevant also for a wide range of other taxa. Moreover, RWAs are perfect flagship species, providing an important example for the establishment of a supranational scheme aimed at the conservation of an invertebrate group. We reviewed the legal aspect of RWA protection and considered how conserving these species must have support in national laws in Europe.

We also reviewed their position on the International Union for the Conservation of Nature (IUCN) Red List, compiled an overview of their protection at the European level, and, finally, found examples representative of the many contradictions and paradoxes that characterize the protection of these species. Our primary aim was to provide a wide overview of RWA protection in Europe by searching information for all the countries entirely included in Europe, with some in‐depth analyses of specific cases that are of general importance because they can be paradigmatic of the difficulties encountered in the protection of many other invertebrate taxa.

## STATUS QUO OF RWA PROTECTION IN EUROPE

### National protection

**FIGURE 1:**
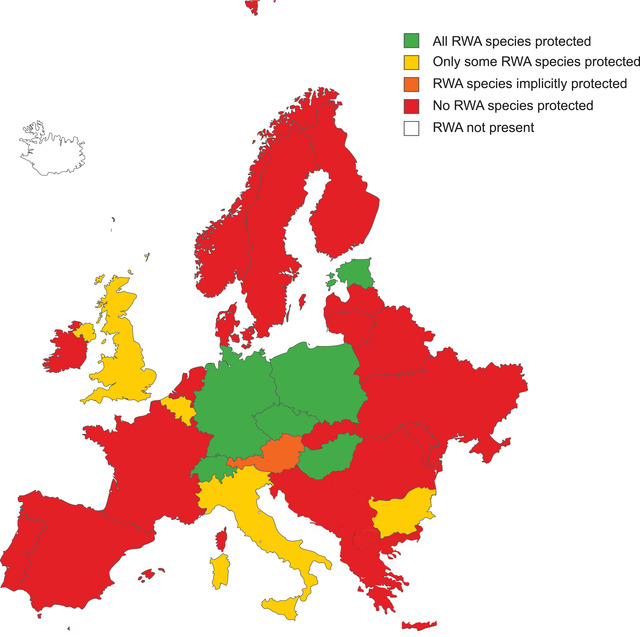
Map of red wood ant (RWA) protection status across European countries.

Several European countries protect RWAs (Figure 1 & Appendix S1). Some of them, such as Austria, implicitly protect them by protecting all wildlife fauna, whereas others explicitly mention RWAs, at least as a group. For example, in Estonia and Poland, all RWAs are protected species, and in Hungary, RWAs are protected and their nests assigned a monetary value. In Switzerland, RWAs are listed as protected since 1966, and all species are explicitly included in Annex 3 of protected species in the Ordinance on the Protection of Nature and Landscape of the Swiss Federal Council. In Germany, besides being protected by the Federal Nature Conservation Act (Bundesnaturschutzgesetz, BNatSchG) like all wildlife, all mound‐building RWAs are additionally listed as specially protected in Germany (like all wild bees and a few wasp species) under the Federal Species Protection Ordinance (Bundesartenschutzverordnung [BArtSchV]), which includes a list of protected species. It is thus prohibited to disturb or destroy their nests or remove workers or other life stages. Moreover, *F. polyctena* x *rufa* hybrids are implicitly protected as well because the parental species are protected. In Belgium, all RWA species were protected by a 1980 law. Later, Belgium legislation was organized at a federal level, and in 2009, the governments of Flanders and Wallonia published a law in which three species in Flanders and two species in Wallonia were protected. Brussels protects only one species (*F. polyctena*). Some other countries explicitly prioritize the protection of particular RWA species. In Bulgaria, some RWA species have been protected since 1959, although the obsolete scientific names included have never been updated, and *F. rufa* is protected by the 2002 Bulgarian Biodiversity Act. In the United Kingdom, *F. pratensis* is a British Action Plan (BAP) 2007 priority species (i.e., those species “identified as being the most threatened and requiring conservation action”) that is also listed in the Species of Principal Importance in England. Also, *F. aquilonia* is included in the Northern Ireland priority species list.

### Italian paradox

Italy is paradigmatic of what happens in the countries at the southern limit of RWA distribution, where less information is available and public awareness is lower. In Italy, these species typically dwell in the Alps (Pavan et al., 1971), where they occur between 450,2000 m elevation. However, this information dates back several decades, and it is considered cautiously because a shift of the distribution area toward higher elevations due to warming, as documented in other insect taxa (Hagen et al., 2007; Moret et al., 2016), is possible. Farther south, the situation is more complex. The only autochthonous species outside the Alps is *Formica pratensis*, occurring also in the Apennine mountains. The actual distribution and abundance of this species are, however, unknown, and the few existing reports are outdated (Pavan et al., 1971), personal observations, sparse, and often unconfirmed notes on citizen science platforms (e.g., iNaturalist). It is clear, however, that some of the Apennine populations have recently disappeared or significantly decreased in number (G. Santini, personal observation). This declining trend is in line with the tendency observed in other countries, such as Switzerland, Belgium, Romania, and Turkey (Çamlıtepe & Aksoy, 2019; Dekoninck et al., 2003; Freitag et al., 2008; Kiss & Kobori, 2010), as well as the British mainland (Nicholson, 1997).

This situation is further complicated by the fact that from the 1950s to the 1980s, several introductions were carried out by transplanting entire RWA nests (mostly belonging to the species *F. paralugubris*) (Masoni et al., 2019) from the Alps to the Apennine mountains as biological control agents (Pavan, 1959). These introductions had varying success. Some populations are developing traits of invasiveness and affecting the native invertebrate fauna (Balzani et al., 2021b; Frizzi et al., 2018) and other taxa (Di Nuzzo et al., 2022).

In Italy, no national law protects RWAs (or any other ant), despite an aborted attempt to include the whole group in a law in 2001 (N. 5013 – Rules for the Protection of the Heterotherm Fauna). Instead, each local authority (region) legislates on the matter. Several regions grant some type of protection (Appendix S2) either by generally protecting ant nests, mentioning the *Fomica rufa* group, or specifying the names of some species (sometimes with misspelled names). Interestingly, one regional law currently grants protection to other ant species, including *Formicoxenus nitidulus*, an obligate myrmecophilous ant listed as vulnerable at a global level (IUCN Red List) that cohabits nests of various RWA species (Härkönen & Sorvari, 2017). Similarly, in the United Kingdom, *F. nitidulus* is a BAP priority species for conservation, but its wood ant hosts are not protected. How to protect an obligate myrmecophile without protecting its host ant is unfortunately not specified.

The most peculiar situation occurs in the regions straddling the Tuscan‐Emilian Apennine, where both the native *F. pratensis* and the introduced *F. paralugubris* occur, the former declining and the latter spreading. Quite surprisingly, protection laws were formulated for the introduced species, and protection started soon after the first introduction in the 1950s (Pavia, Prefectoral Decree 6 April 1956). Moreover, there have been efforts to increase public awareness of the introduced species, whereas the declining *F. pratensis* has not received comparable attention.

### International protection

According to the IUCN Red List (accessed 8 October 2021), RWA species are classified as near threatened at a global level, but only some species (*F. rufa, F. lugubris, F. polyctena, F. aquilonia*, *F. pratensis*, and *F. uralensis*) have been assessed. Previous assessments (from 1983 to 1994) classified all the above RWA species as vulnerable except *F. uralensis*, which was classified as indeterminate (from 1986 to 1994).

The RWAs (and in general, ants) are not included in the European Red List (https://ec.europa.eu/environment/nature/conservation/species/redlist/index_en.htm, accessed 11 October 2021). On the national or regional level, the situation is more complex. Not all European countries include ants, or even insects, in national red lists (https://www.nationalredlist.org/, accessed 11 October 2021). For example, in Ireland, no red list has been produced that covers ants at all, even though all RWA species present are in urgent need of local protection (Breen, 2014). Moreover, when RWAs are considered, there is no consensus across different national red lists on which species to include, assessment criteria differ, some risk categories are not fully comparable, and data deficiency is frequent (Appendix S3). In addition, it is unclear how hybrids, an often‐occurring phenomenon in RWAs, should be treated. For example, only the provisional Red List of the Ants of Flanders explicitly assessed hybrids (*F. rufa* x *polyctena*) (Dekoninck et al., 2003, 2005).

The lack of a comprehensive assessment of the risks faced by RWA species is not surprising because there is a lack of information for all ants. Only 149 out of the approximately 14,000 globally known living ant species (https://www.antweb.org/statsPage.do, accessed 4 January 2022) are listed on the IUCN Red List. For all of them, the last official assessments date back to 1996; thus, an update is needed. RWAs currently face increasing threats throughout their distribution range, but the available information on both threats and distributions is highly variable (Sorvari, 2016). The situation may be particularly critical in the countries at the southern margin of their distribution (Italy, Greece, and Turkey), where the effects of climate change are probably stronger (Rebetez & Reinhard, 2008) and information limited (Kovats et al., 2014). Because in these regions RWAs are restricted to high elevations, the upward shift of populations will progressively be limited by habitat availability. Moreover, only species included in official red lists (following the IUCN criteria) can be protected by law in some countries (e.g., Belgium). Despite their ecological importance, RWA protection receives limited attention, and no effort has been made to standardize protection measures at least in Europe. The complexity of the legal status between and within countries and the diversity of protection measures taken by different states necessitate the development of broad‐scale conservation actions and the deployment of common, coordinated strategies.

## SUGGESTIONS FOR A STRATEGIC APPROACH FOR A FUTURE EUROPEAN CONSERVATION FRAMEWORK

### RWAs as conservation targets

One key decision point is whether to focus conservation efforts on single species or to consider the entire group as a target. Protecting single species has the great advantage of allowing for individually tailored protection policies based on the specific needs of species or local populations. This approach, however, has the associated cost of the harmonization of legal frameworks across countries and requires considerable and informed expertise to support the legal actions. The examples provided here suggest that this is not always the case and that establishing legal protection across the entire group is a far simpler task. Moreover, protection at the species level also faces many difficulties stemming from the taxonomy of these species, starting from the fact that species identification may prove difficult. Furthermore, should hybrids be protected? Hybridization occurs frequently in RWAs and is probably one of the mechanisms promoting speciation (Bernasconi et al., 2011). As pointed out by Robinson and Stockan (2016), conservation measures should allow the preservation of evolutionary processes like this, but how to translate it into laws? Targeting the group could be an easier way to cope with such problems, although care should be taken in distinguishing between autochthonous and introduced species, as the case of *F. pratensis* and *F. paralugubris* in Italy shows.

Moreover, the existence of a law protecting RWAs does not guarantee effective protection because it is often difficult to define what the right protective measures are or should be. When nests are in areas where work is to be carried out (road widening, new construction, etc.), the ant nests are usually moved. Unfortunately, the success rate of these translocations is often low (Serttaş et al., 2020). Forestry practices must also be considered. Even if nests are not directly destroyed during logging, their survival can be hampered by indirect effects resulting from damages to their habitat (Sorvari & Hakkarainen, 2007; Sorvari, 2016). However, these effects should be carefully considered case by case because different species can show different tolerance toward anthropogenic habitat disturbances (Fitzpatrick et al., 2021). In contrast, the natural closure of the forest canopy can eliminate the habitat for RWA species (Vandegehuchte et al., 2017; Fitzpatrick et al., 2021). Viable solutions must, therefore, be proposed to foresters to reconcile logging and the protection of the RWAs. In particular, to achieve effective conservation results, there is the need to train foresters to apply ecologically sound management plans that take into account specific RWA needs on a local basis. Examples are the creation of forest gaps and clearings where canopy closure is excessive or, at the other extreme, reducing the extensions of clearcut areas to facilitate the recolonization of disturbed sites. Also, RWA colony foundation can sometimes rely on temporary social parasitism of colonies of species belonging to the subgenus *Serviformica* (Maeder et al., 2016). The protection of these species could, therefore, facilitate the successful establishment of new RWA colonies.

### RWAs as providers of species‐rich microhabitats

RWAs host many myrmecophiles that thrive in their nest mounds (e.g., Frizzi et al., 2020), some of which are obligate mutualists and cannot survive outside RWA nests (Robinson et al., 2016). Some of these obligate guest species are listed on the IUCN Red List. Clearly, conserving RWAs is integral to protect these organisms, most of which belong to invertebrate groups even less likely to have been assessed for conservation than the Hymenoptera (Parmentier et al., 2014; Robinson et al., 2016). Because the conservation of a species strongly depends on the conservation of its habitat, a thorough revision of the conservation status of myrmecophilous species would be very useful in updating the conservation status of RWAs. Considering RWAs not only as target species but also as providers of species‐rich microhabitats might prove a key strategy to conserve them and all their associated guest species.

## NEED FOR UPDATED INFORMATION ON DISTRIBUTION PATTERNS

Establishing a common and unambiguous legal framework is, however, only the first step toward the effective protection of RWAs. One of the main difficulties in achieving effective conservation strategies is the nonsystematic and sometimes anecdotal information on their distribution, which makes it impossible to monitor populations over time. In turn, the lack of such data hinders the compilation of red lists based on the IUCN criteria. Moreover, habitat requirements are often determined at a local scale from presence‐only data, which can lead to false‐absence biases (but see Vandegehuchte et al., 2017). Switzerland is an important exception; mapping of RWA mounds (especially *Formica lugubris* and *F. paralugubris*) was carried out in the fourth National Forest Inventory (https://www.waldwissen.net/en/forest‐ecology/forest‐fauna/insects‐invertebrates/red‐wood‐ants‐in‐switzerland#c97108). However, these data are incomplete because the sampling design– oriented to trees– did not allow the collection of data suitable for uncommon species, such as *F. rufa* and *F. polyctena*, or species living outside forests, such as *F. pratensis*. Of course, public engagement and citizen science projects contribute greatly to mapping efforts, in particular, because RWA nests are usually conspicuous. Successful cases are the Swiss *Ameisenzeit* (https://www.ameisenzeit.ch/) and *Opération fourmis* (Avril et al., 2019; Freitag et al., 2020), Nest Quest in the United Kingdom (https://www.buglife.org.uk/get‐involved/surveys/nest‐quest/), and the results obtained by Sorvari (2021) in Finland. Furthermore, the activities of amateur associations, such as the *Ameisenschutzwarte* (https://www.ameisenschutzwarte.de/) in Germany, contribute to RWA mapping. However, to enable a European‐level risk assessment, a common, standardized international monitoring strategy for RWA would be vital and would allow the collection of data on RWA habitat requirements in each country. Indeed, RWA occurrence correlates with many environmental features (e.g., Berberich et al., 2016; Vandegehuchte et al., 2017). Furthermore, such a scientifically coordinated monitoring scheme would reduce the inevitable bias related to any survey involving lay organizations. This would finally allow the determination of whether common protection strategies can be applied or more fine‐grained strategies are needed (e.g., between northern and southern countries).

We hope our work will ignite the construction of an international network aimed at the conservation of this important group, at least at the European level.

## Supporting information

Appendix S1. Protection laws and regulations for red wood ant species in European countries.
Appendix S2. Italian regional laws protecting red wood ants.
Appendix S3. Threat status of red wood ant species in Europe according to National Red Lists and local sources.
