# Supplementary material for: Challenges and a call to action for protecting European red wood ants
Source: Conserv Biol. 2022 Aug 5;36(6):e13959. doi: 10.1111/cobi.13959 (PMC10086985; doi:10.1111/cobi.13959)
Supplement: Supplementary file 1 — Appendix S1. Protection laws and regulations for red wood ant species in European countries. Appendix S2. Italian regional laws protecting red wood ants. Appendix S3. Threat status of red wood ant species in Europe according to National Red Lists and local sources. [file COBI-36-0-s001.docx]

**Appendix S1.** Protection laws and regulations for red wood ant species in European countries.

| **Country** | **Reference** | **Comments** |
| --- | --- | --- |
| Austria | NE NSchG, Nature Conservation Act 2000 | Red wood ants are protected because all wildlife is protected |
| Belgium | Royal Law Decree 22 September 1980;  Annex IIIb, M.B. 11th September 1973 and subsequent updates  Flanders: Forest decree and the Nature decree (annex II, III and IV)  Brussels: Ordinance for Nature Conservation (annex II.2 and II.3)  Wallonia: Nature conservation law (annex V) and Directive 2009/147 on the protection of birds (annexes I to VII) | *F. rufa, F. polyctena*, and *F. pratensis* are protected in Flanders.  *F. rufa* and *F. polyctena* are protected in Wallonia |
| Bulgaria | Izvestiya (1959) Order No. 1626 of Ministry of Agriculture and Forestry. No. 101: 2.  Bulgarian Biodiversity Act (2002), Annex 2 and 3. | In the Order No. 1626 of Ministry of Agriculture and Forestry. No. 101: 2, red wood ants are listed but their scientific names have to be updated.  The Bulgarian Biodiversity Act (2002) lists *Formica rufa* as protected |
| Czech Republic | Law on the Protection of Nature and Landscape 114/1992 Coll | *Formica* spp. are listed as threatened in the declaration 395/1992 Col |
| England (in addition to UK regulations) | Species of Principal Importance for England (<http://publications.naturalengland.org.uk/publication/4958719460769792>; last updated 2014). | *F. pratensis* is listed in the "Species of Principal Importance" for England. This qualifies them for protection under the Natural Environment and Rural Communities Act 2006, Section 41, which refers to this list, but doesn't say what species are in it, so that the list itself can be updated periodically.  *F. nitidulus* is also on the Section 41 list |
| Estonia | Regulations of the Minister of the Environment of the Republic of Estonia n. 51, RT I 2004, 69, 1134 | *F. pratensis*, *F. aquilonia*, *F. lugubris*, *F. polyctena*, *F. rufa*, and *F. truncorum* are listed as category III protected species. Interestingly, also *F. nigricans* (currently considered a no more valid species) is listed. |
|  |  |  |
| Germany | Bundesnaturschutzgesetz (BNatSchG; Gesetz über Naturschutz und Landschaftspflege) Artikel 1 des Gesetzes vom 29.07.2009 (BGBl. I S. 2542), in Kraft getreten am 01.03.2010 zuletzt geändert durch Gesetz vom 18.08.2021 (BGBl. I S. 3908) m.W.v. 31.08.2021” (Act on Nature Conservation and Landscape Management, Federal Nature Conservation Act – BNatSchG; Federal Law Gazette 2009, part I, no. 51, p. 2542ff.).  Verordnung zum Schutz wild lebender Tier- und Pflanzenarten (Bundesartenschutzverordnung - BArtSchV) vom 16. Februar 2005 (BGBl. I S. 258, 896), zuletzt durch Artikel 10 des Gesetzes vom 21. Januar 2013 (BGBl. I S. 95) geändert. | Annex I of BArtSchV lists RWA as especially protected species.  *Formica polyctena x rufa* hybrids are implicitly protected. |
| Hungary | Decree 13/2001 v. 9, art. 2 and annex 5 | All red wood ant species are protected. |
|  |  |  |
| Italy | Pavia, Prefectoral Decree 6th April 1956 | Protection addressed to the introduced populations of *Formica paralugubris* but not for the native populations of *Formica pratensis* |
| United Kingdom | Biodiversity Action Plan (BAP) 2007. | The Plan covers *Formica pratensis* but not *Formica rufa, F. lugubris* or *F. aquilonia* which have no general protected status in the UK. Note however that *Formicoxenus nitidulus* is a BAP species, and as it is dependent on *F. rufa* group species, this arguably extends some protection to them. |
| Northern Ireland (in addition to UK regulations) | Wildlife and Natural Environment Act (Northern Ireland) 2011 | *F. aquilonia* is a Northern Ireland Priority Species |
| Poland | Regulation of the Minister of Environment dated December 16, 2016 on the protected species of animals. J. Laws 2016. | All red wood ant species are protected |
| Scotland (in addition to UK regulations) | Scottish Biodiversity List | *Formicoxenus nitidulus* is on the List but none of the *F. rufa* group wood ants are |
| Switzerland | Federal law for the protection of nature and landscape, LPN, RS 451, 1966;  Ordinance on the Protection of Nature and Landscape (OPN), RO 1991-249, 16th January 1991, updated 1st June 2017 | All red wood ant species are protected (Annex 3) |

**Appendix S2.** Italian regional laws protecting red wood ants.

| **Area** | **Region** | **Law Nr** | **Comments** |
| --- | --- | --- | --- |
| Alpine area | Veneto | L.R. 53/1974 | The “*Fomica rufa* group” is mentioned |
|  | Friuli Venezia-Giulia | L.R. 34/1981 | The “*Fomica rufa* group” is mentioned |
|  | Liguria | L.R. 4/1999; L.R. 28/2009 | The “*Fomica rufa* group” is mentioned |
|  | Valle d’Aosta | L.R. 16/1977 | Ant nests in general are protected |
|  | Autonomous province of Trento | L.P. 11/2007 | Ant nests in general are protected |
|  | Piedmont | L.R. 32/1982 | *F. lugubris, F. aquilonia and F. polyctena* are listed, the latter two species with misspelled names |
|  | Lombardy | L.R. 33/1977; L.R. 10/2008 | *F. lugubris, F. aquilonia, F. rufa, and F. polyctena we*re listed, all with misspelled names, in the first law, that was abrogated and substituted by a more comprehensive law that does not mention red wood ants |
|  | Autonomous province of Bolzano | L.P. 27/1973; L.P. 6/2010 | *Fomica rufa* is mentioned, with misspelled name, in the first law, that was abrogated and substituted by a law that does not mention red wood ants |
| Apennines | Umbria | R.R. 1/1981 (abrogated in 2003); L.R. 28/2001 | The “*Fomica rufa* group” is mentioned in the first law, but they are not mentioned in the current regional law |
|  | Lazio | L.R. 4/1999; R.R. 7/2005 | The “*Fomica rufa* group” is mentioned |
|  | Campania | R.R. 3/2017 | The “*Fomica rufa* group” is mentioned. Interestingly, among red wood ants, in Campania only the native *F. pratensis* should potentially occur, although there are no records of this species. |
|  | Tuscany | D.P.G.R. 48/R/2003, Tuscany Forest regulation | The “*Fomica rufa* group” is mentioned. However, the target of this law were the introduced populations of *F. paralugubris* |
|  | Emilia Romagna | General Prescriptions and Forestry Police, approved by regional resolution 182/1995 | The “*Fomica rufa* group” is mentioned. However, the target of this law were the introduced populations of *F. paralugubris* |

**Appendix S3.** Threat status of red wood ant species in Europe according to National Red Lists and local sources.

| **Species** | **Country (Region)** | **Regional status** | | **Assessment year** | | **Criterion** | | **Reference** |  |
| --- | --- | --- | --- | --- | --- | --- | --- | --- | --- |
| *F. polyctena* | Belgium (Flanders) | Vulnerable | | 2003 | | non IUCN | | Dekoninck et al. 2005 |  |
| *F. pratensis* | Belgium (Flanders) | Vulnerable | | 2003 | | non IUCN | | Dekoninck et al. 2005 |  |
| *F. rufa* | Belgium (Flanders) | Vulnerable | | 2003 | | non IUCN | | Dekoninck et al. 2005 |  |
| *F. rufa x polyctena* | Belgium (Flanders) | Indeterminate | | 2003 | | non IUCN | | Dekoninck et al. 2005 |  |
| *F. aquilonia* | Czech Republic | Near threatened | 2017 | | IUCN | | Hejda et al. 2017 | | |
| *F. aquilonia* | Estonia | Least concern | 2017 | | IUCN | | https://elurikkus.ee/lists/public/speciesLists/ | | |
| *F. lugubris* | Estonia | Least concern | 2017 | | IUCN | | https://elurikkus.ee/lists/public/speciesLists/ | | |
| *F. polyctena* | Estonia | Least concern | 2017 | | IUCN | | https://elurikkus.ee/lists/public/speciesLists/ | | |
| *F. pratensis* | Estonia | Least concern | 2017 | | IUCN | | <https://elurikkus.ee/lists/public/speciesLists/> | | |
| *F. rufa* | Estonia | Least concern | 2017 | | IUCN | | <https://elurikkus.ee/lists/public/speciesLists/> | | |
| *F. aquilonia* | Finland | Least concern | 2019 | | IUCN | | Paukkunen et al. 2019 | | |
| *F. lugubris* | Finland | Least concern | 2019 | | IUCN | | Paukkunen et al. 2019 | | |
| *F. polyctena* | Finland | Least concern | 2019 | | IUCN | | Paukkunen et al. 2019 | | |
| *F. pratensis* | Finland | Least concern | 2019 | | IUCN | | Paukkunen et al. 2019 | | |
| *F. rufa* | Finland | Least concern | 2019 | | IUCN | | Paukkunen et al. 2019 | | |
| *F. truncorum* | Finland | Least concern | 2019 | | IUCN | | Paukkunen et al. 2019 | | |
| *F. uralensis* | Finland | Least concern | 2019 | | IUCN | | Paukkunen et al. 2019 | | |
| *F. aquilonia* | Germany | Not threatened | 2011 | | non IUCN | | Seifert 2011 | | |
| *F. lugubris* | Germany | Not threatened | 2011 | | non IUCN | | Seifert 2011 | | |
| *F. paralugubris* | Germany | Not established | 2011 | | non IUCN | | Seifert 2011 | | |
| *F. polyctena* | Germany | Not threatened | 2011 | | non IUCN | | Seifert 2011 | | |
| *F. pratensis* | Germany | Near threatened | 2011 | | non IUCN | | Seifert 2011 | | |
| *F. rufa* | Germany | Not threatened | 2011 | | non IUCN | | Seifert 2011 | | |
| *F. truncorum* | Germany | Threatened | 2011 | | non IUCN | | Seifert 2011 | | |
| *F. uralensis* | Germany | Threatened with extinction | 2011 | | non IUCN | | Seifert 2011 | | |
| *F. aquilonia* | Ireland | Not formally assessed: only one population remains | 2014 | | non IUCN | | Breen 2014 | | |
| *F. lugubris* | Ireland | In serious decline | 2014 | | non IUCN | | Breen 2014 | | |
| *F. aquilonia* | Norway | Least concern | 2021 | | IUCN | | Ødegaard et al. 2021 | | |
| *F. lugubris* | Norway | Least concern | 2021 | | IUCN | | Ødegaard et al. 2021 | | |
| *F. polyctena* | Norway | Least concern | 2021 | | IUCN | | Ødegaard et al. 2021 | | |
| *F. pratensis* | Norway | Least concern | 2021 | | IUCN | | Ødegaard et al. 2021 | | |
| *F. rufa* | Norway | Least concern | 2021 | | IUCN | | Ødegaard et al. 2021 | | |
| *F. truncorum* | Norway | Least concern | 2021 | | IUCN | | Ødegaard et al. 2021 | | |
| *F. uralensis* | Norway | Near threatened | 2021 | | IUCN | | Ødegaard et al. 2021 | | |
| *F. aquilonia* | Poland | Vulnerable | 2002 | | IUCN | | Glowacinski et al. 2002 | | |
| *F. lugubris* | Poland | Vulnerable | 2002 | | IUCN | | Glowacinski et al. 2002 | | |
| *F. polyctena* | Poland | Near threatened | 2002 | | IUCN | | Glowacinski et al. 2002 | | |
| *F. pratensis* | Poland | Near threatened | 2002 | | IUCN | | Glowacinski et al. 2002 | | |
| *F. rufa* | Poland | Near threatened | 2002 | | IUCN | | Glowacinski et al. 2002 | | |
| *F. truncorum* | Poland | Near threatened | 2002 | | IUCN | | Glowacinski et al. 2002 | | |
| *F. uralensis* | Poland | Endangered | 2002 | | IUCN | | Glowacinski et al. 2002 | | |
| *F. aquilonia* | Sweden | Least concern | 2020 | | IUCN | | SLU Artdatabanken. 2020 | | |
| *F. lugubris* | Sweden | Least concern | 2020 | | IUCN | | SLU Artdatabanken. 2020 | | |
| *F. polyctena* | Sweden | Least concern | 2020 | | IUCN | | SLU Artdatabanken. 2020 | | |
| *F. pratensis* | Sweden | Least concern | 2020 | | IUCN | | SLU Artdatabanken. 2020 | | |
| *F. rufa* | Sweden | Least concern | 2020 | | IUCN | | SLU Artdatabanken. 2020 | | |
| *F. truncorum* | Sweden | Least concern | 2020 | | IUCN | | SLU Artdatabanken. 2020 | | |
| *F. uralensis* | Sweden | Least concern | 2020 | | IUCN | | SLU Artdatabanken. 2020 | | |
| *F. polyctena* | Switzerland | Near threatened | 1994 | | non IUCN | | Agosti & Cherix 1994 | | |
| *F. pratensis* | Switzerland | Vulnerable | 1994 | | non IUCN | | Agosti & Cherix 1994 | | |
| *F. rufa* | Switzerland | Near threatened | 1994 | | non IUCN | | Agosti & Cherix 1994 | | |
| *F. truncorum* | Switzerland | Endangered | 1994 | | non IUCN | | Agosti & Cherix 1994 | | |
| *F. uralensis* | Switzerland | Extinct | 1994 | | non IUCN | | Agosti & Cherix 1994 | | |
| *F. aquilonia* | UK | Scarce (Nb) | 1991 | | modified IUCN | | Falk 1991 | | |
| *F. lugubris* | UK | Not a species of conservation concern | 1991 | | modified IUCN | | Falk 1991 | | |
| *F. pratensis* | UK | Endangered: extinct on the mainland; occurs on Channel Islands | 1991 | | modified IUCN | | Falk 1991 | | |
| *F. rufa* | UK | Not a species of conservation concern | 1991 | | modified IUCN | | Falk 1991 | | |

**References**

Agosti D, Cherix D. 1994. Liste rouge des fourmis menacées de Suisse. In: OFEFP (Ed.), Listes rouges des espèces animales menacées de Suisse.

Breen J. 2014. Species dossier, range and distribution data for the hairy wood ant, *Formica lugubris*, in Ireland. Irish Wildlife Manuals, No. 68. National Parks and Wildlife Service, Department of the Arts, Heritage and the Gaeltacht, Ireland.

Dekoninck W, Maelfait JP, Vankerkhoven F, Grootaert P. 2005. Remarks on the distribution and use of a provisional red list of the ants of Flanders (Formicidae, Hymenoptera). JNCC Report 367: 74-85.

Falk S. 1991. A review of the scarce and threatened bees, wasps and ants of Great Britain (No. 35). Peterborough: Nature Conservancy Council for England.

Glowacinski Z, Makomaska-Juchiewicz M, Polczynska-Konior G (Eds.). 2002. Red List of Threatened Animals in Poland. Instytut Ochrony Przyrody PAN, Kraków.

Hejda R, Farkač J, Chobot K. 2017. Červený seznam ohrožených druhů České republiky. Bezobratlí. Příroda, 36, 1-612.

Ødegaard F, Lønnve OJ, Staverløkk A, Sydenham MAK (24.11.2021). Vepser. Norsk rødliste for arter 2021. Artsdatabanken. https://www.artsdatabanken.no/lister/rodlisteforarter/2021/26714

Paukkunen J, Paappanen J, Leinonen R, Punttila P, Pöyry J, Raeknnas M, Teras I, Vepsäläinen K, Vikberg V. 2019. Stinging wasps, bees and ants Aculeata. In: Hyvärinen, Juslén, Kemppainen, Uddström & Liukko (Eds.), The 2019 Red List of Finnish Species. Ministry of the Environment & Finnish Environment Institute, Helsinki.

Seifert B. 2011. Rote Liste und Gesamtartenliste der Ameisen (Hymenoptera: Formicidae) Deutschlands. In: Binot-Hafke, Balzer, Becker, Gruttke, Haupt, Hofbauer, Ludwig, Matzke-Hajek & Strauch (Eds.), Rote Liste der gefährdeten Tiere, Pflanzen und Pilze Deutschlands. Naturschutz und Biologische Vielfalt 70: 469-487.

SLU Artdatabanken. 2020. Rödlistade arter i Sverige 2020. SLU, Uppsala. https://www.artdatabanken.se/globalassets/ew/subw/artd/2.-var-verksamhet/publikationer/31.-rodlista-2020/rodlista-2020.
